# Association between body mass index and tic disorders in school-age children

**DOI:** 10.1186/s12887-024-04592-7

**Published:** 2024-04-20

**Authors:** Lu Bai, Xia Wang, Ruijie Niu, Mengchuan Zhao, Ziwei Zhao, Pengyu Jia, Suzhen Sun

**Affiliations:** 1 Hebei Medical University, Shijiazhuang, 050017 China; 2 Department of Pediatric Neurology, Children’s Hospital of Hebei Province, Hebei Medical University, No. 133 of Jianhuanan Street, YuhuaDistrict, Shijiazhuang, 050031 China

**Keywords:** Tic disorders, BMI, Correlation analysis, Dopamine

## Abstract

**Objective:**

To explore the relationship between body mass index (BMI ) and the severity of tic disorders (TDs) in children 6–14 years old.

**Methods:**

A total of 86 children diagnosed with TDs in a hospital between Jan. 2023 and Sept. 2023 were collected by convenient sampling method, and the general data and TD-specific data were collected and analyzed.

**Results:**

Univariate analysis showed that patients with different Yale Global Tic Severity Scale (YGTSS) grades had statistically significant differences in age, BMI, residence, snacking pattern, weekly physical exercise frequency, weekly physical exercise time, and proportion of cesarean birth. Multiple linear regression analysis showed that the YGTSS score grades were related to BMI, snacking pattern, and cesarean birth of the patients. Correlation analysis revealed a positive correlation between BMI grades and the YGTSS score grades, with a higher BMI indicating more severe TDs. Predictive value evaluation showed that BMI, snacking pattern, and cesarean birth had predictive values for TD severity, and the highest value was found in the combined prediction.

**Conclusion:**

BMI, snacking pattern, and cesarean birth are of predictive values for the severity of TDs. In addition, BMI is positively correlated with the severity of TDs, and a higher BMI suggests more severe TDs.

## Introduction

Tic disorders (TDs) are neurodevelopmental disorders with onset in childhood and adolescence and characterized by irregular, stereotypical motor and/or vocal tics caused by sudden and involuntary contractions of one or more muscles. There are three types of TDs recognized in DSM-5, including provisional tic disorder, chronic tic disorder (CTD) and Tourette syndrome (TS), and CTD is further divided into chronic vocal tic disorder and chronic motor tic disorder based on their clinical features. CTD and TS significantly affect children due to their long disease course, which last more than 1 year, making them the focus of TD studies [1]. In school-age children and adolescents, the prevalences of CTD and TS are 1–2% and 0.3–0.8%, respectively, with a male-to-female ratio of 3:1 [2]. About 40–80% of patients with TDs are complicated with other neuropsychiatric disorders, and attention deficit hyperactivity disorder (ADHD) is the most common one among others [3]. TDs and their complications, especially ADHD, seriously affect the academic performance, quality of life and social communication of TD children [4, 5].

The etiology and pathogenesis of TDs remain unclear. Current studies suggest that the development of TDs is the result of a combination of genetic, immune, environmental, and psychological factors. Structural and functional disturbance of the cortical-striatal-thalamic-cortical (CSTC) circuit, mainly manifested as the abnormal basal ganglia structure and signal transmission, abnormal central neurotransmitter content, the dynamic imbalance of various neurotransmitters, and abnormal neurotransmitter function, is related to the pathology and pathogenesis of TDs [6, 7]. Among them, the dysfunction of dopamine (DA) is believed to be the leading cause of TDs [8]. Furthermore, imaging examinations have shown that some children with TDs have developmental defects and anatomical abnormalities in the central nervous system (CNS), with lesions mainly found in the basal ganglia, frontal cortex, and limbic system [9].

Notably, DA is an important regulator of hypothalamus-mediated autologous food consumption and brain reward circuit-mediated reward-related or hedonic food consumption [10]. There are two hypotheses concerning the role of DA in the development of obesity [11]. In the first hypothesis, food and environment-related signals can activate the reward and pleasure regions of the brain, and result in increased DA release, which is reflected in significantly increased eating behavior and excessive intake of highly palatable foods, leading to obesity. The body satisfies the pleasure through repeated food intake, which leads to the activation of the brain reward system, and the pleasure eventually overrides satiety and hunger, leading to uncontrolled feeding activities and excessive consumption of delicious high-energy food. Food addiction and ultimately obesity occur [12]. Studies have found that obesity and drug addiction can be defined as the same type of reward [13]. The second hypothesis involves a state of reward deficiency, which induces compensatory overeating as compensation for pleasure, and DA reduction in the brain reward circuits is believed to be an important factor for the reward deficiency. Studies have shown that blocked DA receptors (DRs) lead to decreased responses to food motivation, which is very similar to the cancellation or withdrawal of rewards [14].

The evidence suggests a definite correlation between childhood obesity and TDs. However, few studies on the association between childhood obesity and TDs have been reported. Therefore, evaluation of the relationship between the severity of TDs and BMI and further exploration of the possible functional effects and a biochemically coupled relationship between them may provide references for the studies of downstream related neural signaling pathways, with the ultimate goals of guiding clinical practice, provide reasonable and optimized treatment options for children with TDs, and reduce the economic burden of families and society.

## Study subjects and methods

### Study subjects

A total of 86 children diagnosed with TDs in a hospital between Jan. 2023 and Sept. 2023 were selected, and a cross-sectional investigation on the severity of TDs and BMI level was conducted. Inclusion criteria included patients (1) 6–14 years old; (2) met the criteria of the American Psychiatric Association’s *Diagnostic and Statistical Manual of Mental Disorders*, 5th edition [15]; and (3) with no specific changes in EEG and MRI, and no abnormalities in liver and kidney function tests. Exclusion criteria included patients (1) with secondary TDs caused by factors such as genetics, infections, poisoning, and drugs; (2) suffering from rheumatic chorea, hepatolenticular degeneration, epilepsy, and other extrapyramidal diseases; and (3) with tumors (such as leukemia, brain tumors, and reproductive system tumors), severe diseases (such as heart, liver, and kidney diseases), abnormal sexual differentiation (such as blurred genitalia, and cryptorchidism), and long-term use of glucocorticoids. This study was approved by the Ethics Committee of Hebei Children’s Hospital (approval number: 202,335). All participants provided written informed consent before enrollment.

### Methods

The severity of TDs was assessed using the Yale Global Tic Severity Scale (YGTSS), which included the number, frequency, intensity, and complexity of motor and vocal tics.

Physical examination included the standardized height and weight measurements for all children. Standard height and weight scales of T-scale [DZQ-01, Taiwan Heng Precision Measurement and Control (Kunshan) Co., Ltd., China ] were used for measurement, and the instrument was verified and calibrated before use.

The subjects took off their shoes, socks, hats and coats, with only light underwear left. The eye of the observer was on the same horizontal plane as the measuring plate. The accuracy was 0.1 cm for height and 0.1 kg for weight.

BMI was grouped according to the 2017 “American Endocrine Society Clinical Practice Guidelines - Evaluation, Treatment and Prevention of Obesity in Children” [16]. Overweight was defined as a BMI of P85 - < P95, obesity as a BMI ≥ P95, and normal as a BMI < P85 in the growth curve standard. BMI was calculated as weight (kg)/height (m)^2^.

In YGTSS [17] scoring, the global YGTSS score had a range of 0-100, and TDs were graded as mild, moderate, and severe as the global YGTSS score < 25, 25–50, and > 50, respectively.

Measurement tools were centrally purchased. Questionnaire survey data were entered by professionals, double-checked, and subjected to initial, second, and final reviews. Data that passed all reviews were considered valid.

### Data collection

General data and the YGTSS scores of the patients were collected. General data included family monthly income, duration of breastfeeding, average night snack frequency, Western fast-food frequency, snacking pattern, weekly physical exercise frequency, weekly physical exercise time, daily time spent using electronic products, daily sleep time, age, residence, and BMI.

### Statistical analysis

SPSS 26.0 statistical software was used for statistical analysis, and the K-S method was used for the normality test. Measurement data with normal distribution were represented as (x ± s), and mean values were compared among groups using analysis of variance; data with skewed distribution or rank data were compared among groups using Kruskal-Walis H test; enumeration data were represented as frequency (n) or rate (%), and χ^2^ test was used for comparison; dichotomous variables were analyzed using multiple linear regression for multivariate analysis; the predictive values of various factors on the severity of TDs were explored using the receiver operating curve (ROC); and Pearson correlation analysis was used for correlation analysis. A significance level of α = 0.05 was used for all tests.

## Results

### Comparison of the YGTSS score grades for general data of different patients

The results showed that there were 30 mild TD patients, including 13 boys and 17 girls, with an average age of 6.98 ± 2.25 years, 31 patients with moderate TD, including 15 boys and 16 girls, with an average age of 8.14 ± 2.07 years, and 25 patients with severe TD, including 9 boys and 16 girls, with an average age of 8.50 ± 2.12 years. Statistically significant differences were observed in age, BMI, residence, snacking pattern, weekly physical exercise frequency, weekly physical exercise time, and proportion of cesarean birth among the three groups(*p* < 0.05). There were no statistically significant differences in primary site of TDs, sex, course of disease, monthly family income, duration of breastfeeding, average night snack frequency, Western fast-food frequency, average daily time spent using electronic products, and daily sleep time among the three groups of patients (*p* > 0.05)(Table 1).

**Table 1:** Comparison of the YGTSS score grades for general data of different patients

items	mild(*n* = 30)	moderate(*n* = 31)	severe(*n* = 25)	F/χ^2^/Z	P
age	6.98 ± 2.25	8.14 ± 2.07	8.50 ± 2.12	4.139	0.012
primary site of TD			25	0.460	0.795
facial	18	21	15		
non-facial	12	10	10		
BMI	19.95 ± 4.75	24.96 ± 4.38	24.55 ± 6.43	10.724	< 0.001
sex(M/F)	13/17	15/16	9/16	0.868	0.648
course of disease	4.56 ± 5.87	5.64 ± 3.70	6.10 ± 4.78	2.469	0.091
residence				6.987	0.030
urban	15	19	21		
rural	15	12	4		
family monthly income(CNY)				0.142	0.931
< 3000	2	3	2		
3000–5999	9	8	8		
6000–8999	13	14	11		
9000 -	6	5	4		
duration of breastfeeding(months)	11.35 ± 4.14	12.87 ± 3.14	13.65 ± 4.57	0.560	0.715
average night snack frequency	30			4.820	0.090
once every 1–2 days	7	11	10		
once every 2–3 days	8	11	10		
once every 4 days or less	15	9	5		
Western fast-food frequency				3.783	0.151
once every 1–2 days	5	7	9		
once every 2–3 days	7	8	7		
once every 4 days or less	18	16	9		
snacking pattern				6.834	0.033
high-sugar and high-calorie	11	16	18		
other	19	15	7		
weekly physical exercise frequency				6.168	0.046
Once every 1–3 days	14	10	6		
Once every 4–6 days	12	13	8		
Once a week or less	4	7	11		
weekly exercise time (h)				10.727	0.005
< 1	4	9	11		
1 -	7	8	7		
4 -	8	8	6		
> 7	11	6	1		
average daily time spent using electronic products(h)				0.053	0.974
< 1	2	1	1		
1–3	11	12	10		
> 3	17	18	14		
daily sleep time (h)				3.453	0.178
< 8	4	7	11		
8-	15	14	7		
> 10	11	10	7		
caesarean birth (n)		31		11.519	0.003
yes	4	13	14		
no	26	18	11		

### Multivariate analysis of factors influencing the grading of YGTSS scores

To further explore the factors influencing the grading of YGTSS scores in children, stepwise regression analysis was conducted using the YGTSS score grades as the dependent variable, and the variables with statistically significant differences in univariate analysis as independent variables.

Factors with differences in the univariate analysis were assigned values as follows: residence: urban = 1, rural = 2; snacking pattern: high-sugar and high-calorie = 1, other = 2; weekly physical exercise frequency: once every 1–3 days = 1, once every 4–6 days = 2, once a week or less = 3; weekly physical exercise time: <1 = 1, 1- = 2, 4- = 3; caesarean birth: yes = 1, no = 2; and the remaining variables were included as continuous variables. The stepwise regression method was used to include and exclude the independent variables (*α*_entry_ = 0.05, *α*_removal_ = 0.1), and the influencing factors with interactions were eliminated. The results showed that age, residence, weekly physical exercise frequency, and weekly physical exercise time were excluded in the process of inclusion and exclusion of variables, and the resulting regression model R^2^ was 0.734, indicating that BMI, snacking pattern and proportion of cesarean birth could explain 73.4% of the YGTSS score grading, *F* = 123.73, *p* < 0.001. This suggested that the dependent variable YGTSS score grades fitted well with the three independent variables, with a Durbin-Watson index of 1.995. Therefore, there was no correlation between the independent variables of the model. The significance tests of the three independent variables in the model showed p values less than 0.05, which proved that the three independent variables were statistically significant in the model and should be retained. In addition, the VIF values of the three independent variables were far less than 10, indicating no collinear relationship between the variables. The multiple linear regression equation obtained after fitting was as follows:

Y = 4.314 + 0.412 × _1_ + 0.342 × _2_ + 0.355 × _3_.

According to the partial regression coefficient in the model, it was concluded that the degree of influence of these 4 independent variables on the grading to YGTSS scores was BMI > cesarean birth > snacking pattern(Table 2).

**Table 2:** Regression model for factors influencing the grading of YGTSS scores

variables	standard error	partial regression coefficient	P	95%CI	VIF
constant	4.314	-	<0.001	-	-
BMI	1.319	0.412	<0.001	0.154 ~ 0.462	1.057
snacking pattern	1.612	0.342	<0.001	0.234 ~ 0.432	1.055
caesarean birth	1.192	0.355	<0.001	0.221 ~ 0.435	1.087

Note: *R*^*2*^ = 0.734, adjusted *R*^*2*^ = 0.643, *F* = 123.73, *P* < 0.05

### Correlation analysis between BMI grades and the YGTSS score grades

Pearson correlation analysis was used to explore the relationship between BMI grades and the YGTSS grades, and it was found that BMI grades were positively correlated with the YGTSS grades (*r* = 0.261, *P* = 0.015), and the severity of TDs generally increased with the increase of BMI grades(Table 3).

**Table 3:** Correlation analysis between BMI grades and YGTSS score grades

variables	mild	moderate	severe
normal	15	12	5
overweight	9	10	9
obesity	6	9	11

*r* = 0.261, *P* = 0.015

### Predictive value of each indicator for the severity of TDs

The results showed that BMI, snacking pattern and caesarean birth had predictive values for the severity of TDs. The area under the curve (AUC) for predicting the severity of TDs was 0.693 (95%CI 0.671–0.756) for BMI, 0.656 (0.619–0.754) for snacking pattern, and 0.661 (0.625–0.738) for caesarean birth. The highest predictive value was found in the combined prediction, with an AUC of 0.771 (0.714–0.811) (Table 4).

**Table 4:** Predictive value of the indicators for the severity of TDs

items	AUC	95%CI	cutoff	sensitivity (%)	specificity (%)
BMI(kg/m^2^)	0.693	0.671 ~ 0.756	26.56	81.31	74.54
snacking pattern	0.656	0.619 ~ 0.754	-	80.61	81.70
caesarean birth	0.661	0.625 ~ 0.738	-	82.01	63.82
combined prediction	0.771	0.714 ~ 0.811	-	86.24	87.73

## Discussion

TDs are divided into three types according to DSM-5 [18], including provisional tic disorder, chronic tic disorder, and Tourette syndrome. The maximum severity of tics often occurs at the age of 10–12 years, and the severity decreases from adolescence to early adulthood [19]. TDs, especially provisional TDs, are relatively common in childhood, with a reported prevalence of 6–12%. Epidemiological studies have shown that the prevalence of TS in children is about 1%, and nearly half of the cases will continue until adulthood [20]. Most patients with TS have comorbidities, such as ADHD, mood disorders, and obsessive-compulsive disorder(OCD).

The psychological stress, smoking, alcohol consumption, and diseases in pregnant women during pregnancy, as well as fetal asphyxia, amniotic fluid inhalation, premature delivery, and post-term delivery may lead to an increased incidence of TDs in children [21]. Due to the fact that the first trimester of pregnancy is a critical period for the development of the fetal nervous system, adverse conditions such as emotional disorder, malnutrition, and threatened miscarriage during this period affect the development of the fetal brain, leading to impaired communication pathways between the brain and the basal ganglia, cortex, thalamus, and midbrain, as well as disordered amygdala striatum pathways, and consequent behavioral and motor disorders [21]. Studies have shown that factors such as prenatal (such as alcohol abuse, and smoking), perinatal (such as streptococcal infections, and complications), and birth environment can induce the development of TDs through various ways, including DNA methylation [21]. In addition, studies have also reported that maternal smoking during pregnancy increases the severity of TS in children [22], suggesting that adverse factors during the perinatal period and birth often lead to brain damage and impaired nervous system development in children, which reduces the children’s resistance to other risk factors during their growth is reduced and leads to diseases. The results of the present study showed that cesarean birth is a major risk factor for severe TD, as reflected in the increased proportion of cesarean births in the group of patients with severe TDs, which is consistent with the findings described above.

A currently well-recognized hypothesis is that the etiology of TDs involves the disorder of the dopamine system. Although the specific neuropathological mechanism of TDs remains unclear, there is a large amount of evidence regarding the relationship between the dopamine system and TDs, including dopamine dysfunction or metabolic abnormalities in the prefrontal lobe, striatum, and other areas [23]. Serra-Mestres et al. [24] compared 10 patients with TDs and 10 normal controls using SPECT technique and found that the density of dopamine transporters in the caudate nucleus and putamen of the TD group was higher than that in the control group, suggesting that abnormalities in dopamine transporters in the caudate nucleus and putamen are involved in the pathogenesis of TDs. Minzer et al. [25] determined the densities of dopamine receptors (D1, D2), transporters, vesicular monoamine transporter protein II and vesicle release and recycling proteins, and α2 adrenergic receptors in the caudate nucleus, putamen nucleus, ventral striatum and prefrontal cortex of the corpse of patients with TDs using semi-quantitative immunoblotting, and found that the densities of dopamine D2 receptors, transporters, and α2 adrenergic receptors were significantly increased in the prefrontal lobe, rather than in the striatum, of patients with TDs, and the density of dopamine D2 receptors in the prefrontal lobe of all TD patients was about 140% higher than that of the control group. Genes involved in the metabolic pathways of the dopaminergic system have become the candidates for TDs, and dopamine receptor genes have undoubtedly become the focus of studies, with the dopamine D4 receptor gene as the most attractive one.

The dopamine D4 receptor is mainly distributed in the frontal cortex, midbrain, amygdala and medulla oblongata, with less distribution in the neostriatum, and primarily mediates the postsynaptic activity of dopamine. In recent years, the dopamine D4 receptor has been regarded as an important research focus for mental illness closely related to dopamine function, and many studies have suggested that TDs, ADHD, OCD, schizophrenia, and personality disorder is related to dopamine D4 receptor gene polymorphism [26]. These studies mostly focused on the polymorphism of the 48 bp VNTR in exon 3 of the dopamine D4 receptor gene. Grice et al. [27] analyzed the polymorphism of the 48 bp repeated sequence in exon 3 of the dopamine D4 receptor gene in 64 probands and their biological parents, and found linkage disequilibrium in the allele DRD4·7R. Curz et al. [28] found that the frequency of allele A7 was 91% in OCD patients with TDs and 41% in OCD patients without TDs, and the difference was statistically significant, indicating that the allele DRD4.7R is a risk factor for OCD patients complicated with TDs. Diaz-Anzaldua et al [29] conducted a transmission disequilibrium test (TDT) and haplotype analysis on 110 French-speaking Canadian families with TDs, and found a significant correlation between alleles and TDs.

Dopamine dysfunction may induce the body to satisfy the sense of pleasure through repeated intake of food, resulting in the activation of the brain reward system. The pleasure eventually overrides the satiety and hunger, leading to uncontrolled feeding activities and excessive consumption of delicious high-energy food. Food addiction and ultimately obesity occur [12]. The results of the present study showed that individuals with severe TDs are more likely to consume high-sugar and high-calorie food, with higher BMI, which was consistent with the findings above.

Our study has several limitations that should be acknowledged. Firstly, this was a single-center study, and the sample size was small with poor representativeness. Therefore, our findings may not be generalizable to a broader population of children with TDs. Future studies should use larger and more representative samples to validate our findings. Secondly, the use of a convenience sampling method may introduce bias, as the participants may not be randomly selected from the target population. A more randomized approach is suggested for future research to reduce the selection bias and increase the internal validity of the study. Thirdly, the absence of a control group of children without tic disorders is a significant limitation, as it restricts the ability to determine if the observed associations are unique to tic disorders or applicable more broadly to the pediatric population. However, we did not have access to such a group in our study setting, and we could not randomly assign children to different groups due to ethical reasons. Therefore, we used the YGTSS score grades as a proxy for the severity of tic disorders, and compared them among different groups of children with different BMI levels, snacking patterns, and cesarean births. We have clarified this point in the methods section. Fourthly, the cross-sectional nature of the study limits the understanding of the causal relationship between BMI and tic disorders. Longitudinal studies are recommended for future research to better comprehend this relationship’s direction and dynamics.

## Conclusion

In summary, BMI, snacking pattern, and cesarean birth are of predictive values for the severity of TDs. In addition, BMI is positively correlated with the severity of TDs, and a higher BMI suggests more severe TDs. Intervention should be promptly taken to reduce weight for TD children with a high BMI. Our study is one of the few studies that investigated the association between BMI and the severity of TDs in children, and provided evidence for the link between dopamine dysfunction and both TDs and obesity. Our study also identified BMI, snacking pattern, and cesarean birth as predictive factors for the severity of TDs in children, and provided a simple and practical tool for screening and intervention. Our study has implications for the clinical practice, policy making, and education of TDs and obesity in children, and may help improve the diagnosis, treatment, and prevention of these conditions. Our study also has implications for the understanding of the etiology and pathogenesis of TDs and obesity, and may provide new insights and directions for future research.

## Data Availability

No datasets were generated or analysed during the current study.
